# Linen Leagues for Hospitals

**Published:** 1916-04-01

**Authors:** 


### THE EDITOR'S LETTER-BOX.

**Our Correspondents are reminded that brevity of style and conciseness of statements greatly facilitate early insertion. We cannot in any way be responsible for the opinions expressed by correspondents, who must give their name and address as a guarantee of good faith, but not necessarily for publication.**

### Linen Leagues for Hospitals.

*To the Editor of* The Hospital.

Sir,—With reference to Mrs. Grant's statement at the meeting of the Plymouth Linen League, surely there must be some error; it is there stated that 4,000 articles were washed weekly, that is 208,000 yearly, at a cost of £738, or something under 1¼d. each. Many of those articles are large, such as blankets, quilts or counterpanes, sheets, and soiled and stained to such an extent that it is most difficult—in fact, frequently impossible—even with strong and expensive acids to completely remove such stains at a cost of over 1¼d. each; but however much it may be desirable for each hospital to have a laundry capable of doing its own washing, it is equally desirable that the question should not be placed upon a false basis. To endeavour to make it appear that there could be any saving by spending thousands of pounds upon buildings and machinery, several items of which would cost nearly as much as is paid for the year's work, while much of the machinery would only last a few years before it required to be replaced, to say nothing of the materials and labour, is asking too great a credence from the charity and good nature of the public who would have to find the cash to carry out the idea.

My experience of and interest in both systems is that it would be a great mistake to go begging for means to build and equip a large laundry for any hospital at the present time; there are dozens of laundries now to be sold. The cost of materials to build, etc., to carry on and maintain a laundry has doubled in price since the war began; besides, wages are higher and the difficulty in obtaining labour is increasing.

The managers of hospitals would act wisely to weigh all the pros and cons before making any appeal to the public for cheques for such purpose.—Yours truly,

Sedgefield.

[*** Our correspondent's arithmetic is faulty, the cost per piece would be about four-fifths of a penny. This is a rate rather less than that at the first hospital, whose report we have consulted, doing its washing on the premises, and about the same as another large institution sending its washing out. But an important consideration —that of the wear and tear and loss of articles washed is not dealt with in the above letter, and the question as to whether these would be less in a hospital than a public laundry must influence the authorities at Plymouth in coming to a decision just as much as the cost of washing. —Ed. The Hospital.]

